# Ultrasound and Photoacoustic Imaging for the Guidance of Laser Ablation Procedures

**DOI:** 10.3390/s24113542

**Published:** 2024-05-30

**Authors:** Samuel John, Yan Yan, Shirin Abbasi, Mohammad Mehrmohammadi

**Affiliations:** Imaging Science, University of Rochester Medical Center, Rochester, NY 14642, USA; samjohn.jk@gmail.com (S.J.); yan_yan1@urmc.rochester.edu (Y.Y.); shirinabbasi.md@gmail.com (S.A.)

**Keywords:** ultrasound, photoacoustic, thermometry, tracking, laser, lesion, monitoring, ablation

## Abstract

The accuracy and efficacy of laser ablation procedures depend on the accurate placement of the laser applicator within the diseased tissue, monitoring the real-time temperature during the ablation procedure, and mapping the extent of the ablated region. Ultrasound (US) imaging has been widely used to guide ablation procedures. While US imaging offers significant advantages for guiding ablation procedures, its limitations include low imaging contrast, angular dependency, and limited ability to monitor the temperature. Photoacoustic (PA) imaging is a relatively new imaging modality that inherits the advantages of US imaging and offers enhanced capabilities for laser-guided ablations, such as accurate, angle-independent tracking of ablation catheters, the potential for quantitative thermometry, and monitoring thermal lesion formation. This work provides an overview of ultrasound-guided procedures and how different US-related artifacts limit their utility, followed by introducing PA as complementary to US as a solution to address the existing limitations and improve ablation outcomes. Furthermore, we highlight the integration of PA-driven features into existing US-guided laser ablation systems, along with their limitations and future outlooks. Integrated US/PA-guided laser ablation procedures can lead to safer and more precise treatment outcomes.

## 1. Introduction

Image guidance is paramount in medical interventions, similar to navigational technologies employed to navigate vehicles through unknown terrain [1]. Image-guided surgical procedures utilize the demarcation of anatomical features, such as tissue vasculature, to benefit surgical efficacy and prevent or minimize collateral tissue damage. To date, image-guided procedures have been widely used in various clinical and pre-clinical applications in which an external therapeutic or assistive device can be accurately navigated within the tissue [2,3,4,5]. Image guidance in surgeries reduces the number of recurrences, the duration of hospitalization, and the probability of accidental damage to proximal non-target tissues. The utility of non-invasive imaging in image-guided surgeries has enabled the transition from open surgery settings involving the direct identification and sense of organs to image-based feedback for better control and optimizing the procedure outcome [6]. Image-guidance systems aim to augment and aid the surgeon in understanding the spatial anatomical structure, as well as provide complementary information that provides feedback on the efficacy of the procedures.

Laser ablation has been used in various medical applications, from treating atherosclerotic plaques to its current use in oncology, lithotripsy, and vascular therapies [7]. A wide range of laser wavelengths ranging from λ = 350–10,600 nm are employed in clinical procedures because the absorption spectra of tissue chromophores depend on the wavelength of the incident photon energy, resulting in its destruction [8]. Laser ablation has been used for treating superficial gastrointestinal cancers, colorectal adenoma, and high-grade Barrett’s esophagus [9]. Laser lithotripsy has been used to obliterate urinary calculi through photothermal or photomechanical effects. The short-pulsed dye laser (λ = 504 nm) treats cystine stones due to the absorption of laser light, followed by a photomechanical effect induced within the yellow-colored urinary stones leading to their fragmentation [10]. The long-pulsed Holium:YAG laser (λ = 2100 nm) is used for treating multiple calculi through the photothermal effect caused by the absorption of laser light by water, inducing vapor and destroying the surrounding kidney stones [11,12]. Endovenous laser ablation utilizes a λ = 1470 nm, continuous-wave laser tuned to the absorption spectra of water molecules present in the vein wall for treating varicose veins [13].

**Figure 1:**
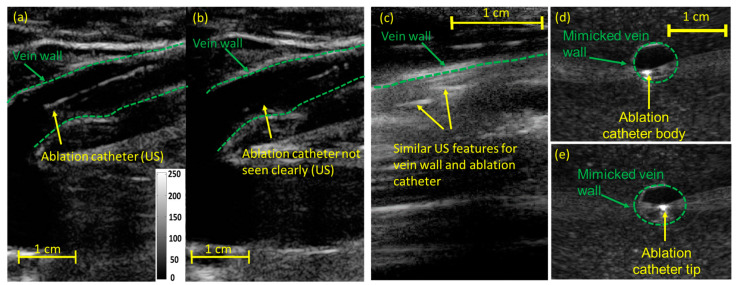
Limitations of US imaging in guiding laser ablation procedures: (**a**) US imaging guides the ablation catheter through the veins in EVLA procedures. Due to angular dependency or catheter–transducer misalignment, (**b**) US imaging fails to visualize the catheter tip within the vein. The image (**a**,**b**) was adapted from [28]. (**c**) The poor CNR of US images fails to distinguish the catheter within the tissue background. Due to similar US features shared between the catheter tip and body, (**d**) US imaging fails to differentiate between (**d**) the catheter body and the (**e**) tip.

Ultrasound (US) imaging aids in guiding clinical procedures such as the placement of brachytherapy radiation sources for treating cancer [14,15], ablation catheters [16,17,18] (Figure 1a) for vascular therapies, needles [2,3,19,20,21,22], and stents [4,5,23,24] in real time. US-guided catheter tracking is limited due to insufficient contrast, difficulty determining the ablation catheter tip, and angular dependency (Figure 1b). Angular dependency is characterized by the inaccurate catheter tip [16,25,26,27,28] localization caused by the reflected sound waves moving away from the imaging plane and not being received by the transducer [29]. The sub-optimal contrast to noise ratio between the catheter and the background tissue limits the capabilities of US imaging to detect the catheter [30] (Figure 1c). Moreover, due to the similar US appearance of the catheter body and the tip, US imaging fails to distinguish between them (Figure 1d,e), inducing false tip positioning-based inaccuracies. In addition, US artifacts, including reverberation [31] and comet tail artifacts [32], limit the accurate positioning of the catheter tip within the tissue. US imaging has also been used to monitor the outcome of laser ablation procedures by monitoring the temperature during the procedure [33] and the formation of thermal lesions [34]. However, boiling and cavitation during thermal therapies induce US artifacts such as acoustic shadowing, which limits the US’s utility in thermometry [35]. Since US thermometry techniques are highly tissue-dependent, US is often limited to temperatures below 50 °C [35]. Moreover, the US-based thermometry technique above 50 °C is limited by the non-linear temperature dependencies of contrast parameters, tissue phase transitions, and cavitation bubbles [35]. In addition, the ability of the US to monitor the extent of the thermal lesion is not yet proven to be very accurate and valuable for clinical applications as it primarily relies on tissue echogenicity and faces difficulties in hyperechoic and heterogeneous tissues [34,36].

**Figure 2:**
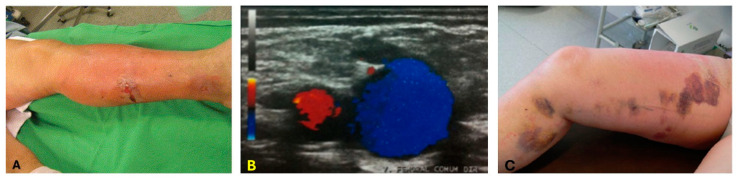
Limitations of EVLA without proper thermometry. These limitations include (**A**) skin burns. The image was adapted from [41]; (**B**) endovenous heat-induced thrombosis (EHIT). The image describes the thrombus flow within the vein. The flow of thrombus moving toward the transducer is color-coded in red, and blue indicates the flow away from the transducer. The image was adopted from [42]; (**C**) ecchymosis. The image was adapted from [43].

A lack of non-invasive thermometry features leads to an uncontrolled dose, which induces overheating in the surrounding tissue. In the case of EVLA, for example, the overheating of the perivenous tissues causes skin burns (Figure 2A) and thrombosis (Figure 2B). High peak temperatures within the vein perforates the vein wall, inducing more perivenous damage, causing more pain and ecchymosis [37,38,39] (Figure 2C). In some cases, a lack of control over the thermal dose deposition may lead to the delivery of an insufficient thermal dose inducing the recurrence of varicose veins. In some clinical laser ablation procedures, such as Twin-to-Twin Transfusion syndrome, the low-contrast images due to the scattering of light in the endoscope [40] and the lack of quantitative information in the images limits its ability to confirm the extent of ablation. The effectiveness of laser ablation procedures depends on the accurate placement of the laser applicator within the diseased tissue and monitoring the real-time temperature variations during the ablation procedure.

Within the rapidly growing novel medical imaging domain, photoacoustic (PA) imaging has gained significant traction and popularity due to its unique features, including image contrast, facile integration with US systems, and non-ionizing in nature. The PA signal’s dependence on chromophores’ absorption properties makes PA imaging highly sensitive in detecting tissue chromophores. Using a US transducer to acquire PA signals promotes easy adoptability into procedures. Unlike X-ray fluoroscopy, PA imaging utilizes non-ionizing laser pulses for tissue excitation. PA imaging, also known as optoacoustic imaging, is a non-ionizing modality that produces US waves generated from nanosecond-pulsed light absorption and the subsequent thermoelastic expansion of a specific tissue chromophore [44,45]. PA imaging can provide functional information like blood flow [46], temperature [47], and blood oxygenation [48]. Laser-induced PA acoustic waves are spherical and travel omnidirectionally from the source. The US transducer picks up these acoustic waves and forms the final image. Compared to US images, which characterize the mechanical and elastic properties of the tissue [49], a PA image characterizes the optical properties, especially the optical absorption [44,45,50]. Besides providing functional and molecular information about the imaged tissue, PA imaging can be used for real-time catheter tip tracking because the PA signal is generated at the interface between the fiber and the tissue. The temperature-dependent PA amplitude allows for real-time thermometry [28,47,51].

## 2. Ultrasound and Photoacoustic Imaging for Guidance of Laser Ablation Catheters

**Figure 3:**
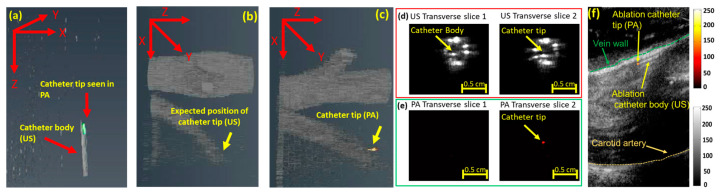
PA-guided catheter tip tracking: (**a**) volumetric US/PA image highlighting the catheter tip seen using PA imaging and the catheter body revealed using US imaging. (**b**) Due to angular dependency, US imaging cannot accurately locate the catheter tip within the angled vein. However, (**c**) PA imaging detects the catheter tip. The image was adopted from [28]. Unlike (**d**) US imaging, which fails to distinguish between the catheter tip and the body, (**e**) PA imaging distinguishes them. The image was adapted from [28]. (**f**) PA imaging provides high-contrast images of the catheter tip superimposed on the anatomical structure of the US image. Images were adapted from [47].

During image-guided ablation therapies, PA imaging can be used for real-time catheter tip tracking because the PA signal, generated at the tip of the catheter where the laser light interacts with the tissue, generates PA acoustic waves that travel omnidirectionally and can be picked up with a US receiver with no limitation forced by angular dependency. The generated PA signal is described as $P=\Gamma \times \mu_{a}\times F$, where Γ corresponds to the Gruneisen parameter, µ_a_ is the absorption coefficient, and laser fluence is denoted by F. Our earlier studies previously showed PA-guided catheter tip tracking [28,51] in which vessel-mimicking polyvinyl alcohol-based and ex vivo tissue-based phantoms were imaged. Volumetric US and PA images of the 532 nm coupled catheter within vessel-mimicking phantoms resembling the catheter’s position in two different orientations revealed that US imaging visualizes the catheter body and PA imaging detects the accurate location of the catheter tip (Figure 3a) [28]. While US imaging loses the catheter tip (Figure 3b), PA imaging provides angle-independent images of the catheter tip (Figure 3c) [51]. US and PA images can be easily superimposed onto each other as they are naturally co-registered. Similar experiments have been conducted to highlight the catheter tip-tracking capabilities of PA imaging [16,28]. Figure 3d,e highlight the ability of PA imaging to distinguish between the catheter tip and the body. US imaging fails to distinguish between the catheter tip and the body due to the similar features shared between them (Figure 3d). High-contrast PA-images of the catheter tip can be acquired by tuning the laser pulses to the absorption spectrum of the medium surrounding the catheter tip (Figure 3e) [52,53]. In addition, PA images of the catheter tip indicated an SNR of 6.598 and CNR of 6.130 with a low-pulse energy of 100 µJ. These results and the CNR values support the feasibility of guiding EVLA catheters in blood vessels using a low-energy pulsed laser.

An in vivo study featuring the pulsed laser-coupled ablation catheter within a dog’s jugular vein further validated the capabilities of PA catheter tracking. US imaging fails to detect the catheter tip due to the poor CNR between the catheter and the anatomical structure of the tissue. Moreover, the similar structures shared between the catheter body and the vessel add further inaccuracies. PA imaging provides high-contrast, artifact-free images of the catheter tip (Figure 3f). PA images of the catheter tip superimposed on the anatomical US background image allow for accurate catheter tip visualization and placement, thus potentially improving the procedure’s outcome.

## 3. Photoacoustic Thermometry during Laser Ablation

During image-guided laser ablation surgeries, concurrent monitoring of the tissue temperature and controlling the power of the ablation laser play a paramount role in treatment, alongside the placement of the catheter tip in the desired location. Existing image-guided ablation systems fail to monitor the localized tissue temperature and deliver a blind treatment by inducing an undesired thermal dose, which causes ecchymosis, perivenous tissue damage, deep vein thrombosis, and, sometimes, the recurrence of varicose veins. PA imaging exploits the dependence of the Gruneisen coefficient, a measure of the efficient conversion of heat energy to pressure, to non-invasively monitor the real-time temperature change in tissues [45]. The Gruneisen coefficient is defined as $\Gamma =\frac{{\beta c}^{2}}{C_{p}}$, where β is the thermal coefficient of volume expansion, *c* corresponds to the speed of sound, and C_p_ is the heat capacity at constant pressure. The Gruneisen parameter depends on the speed of sound. Hence, the PA signal and the Gruneisen parameter amplitudes are temperature-dependent. PA thermometry is based on the principle that variations in ambient temperature induce a change in the PA signal amplitude. This principle is defined as $\Delta T=a\frac{\Delta P}{P}F$, where $\frac{\Delta P}{P}$ refers to the relative PA signal amplitude change, ‘a’ refers to the temperature-dependent constant, ∆T refers to the ambient temperature variations, and laser fluence is denoted by F.

**Figure 4:**
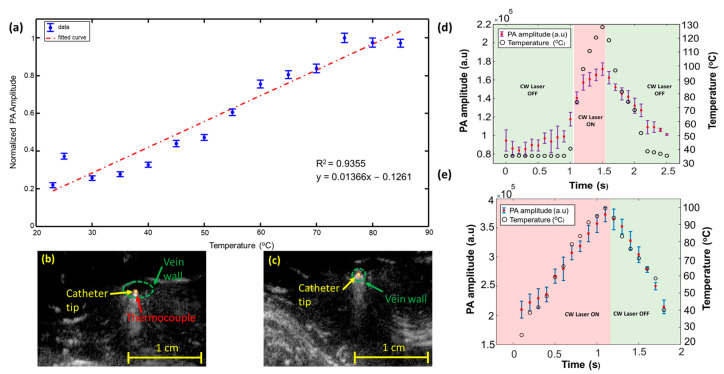
Real-time PA thermometry: (**a**) a clear increment in the PA signal with temperature variations is observed within a water-filled US transparent tube. The linear fit highlights the normalized PA amplitude, and the blue dots indicate measurements. The image (**a**) was adapted from [28]. Co-registered US and PA images highlight the placement of the ablation catheter tip and the K-type thermocouple (**b**) suspended in the vein’s lumen and (**c**) squeezed against the vein wall. The images (**b**,**c**) were adapted from [47]. (**d**) Variation in the PA amplitude generated at the ablation catheter tip placed within the lumen of the vein due to the temperature variations induced by the CW ablation laser energy. (**e**) Changes in the PA signal amplitude generated at the ablation catheter tip squeezed against the vein’s wall due to the high peak temperature variations caused by the CW ablation laser energy. Images (**d**,**e**) were adapted from [47].

The potential of real-time PA thermometry was validated through ex vivo and in vivo animal studies. The real-time temperature variations at the catheter tip were monitored by acquiring PA images of the ablation catheter tip placed within a water-filled US-transparent tube, whose temperature was varied between 23 and 85 °C at increments of 5 °C. The increase in the PA signal amplitude [28,51] (Figure 4a) can be accounted for by the increase in the Gruneisen parameter of water at higher temperatures. Further in vivo studies using a combined beam (532 nm pulsed and 1470 nm CW laser) in the canine jugular vein confirmed PA thermometry’s abilities. Given that the temperature monitoring in water-based tissue such as vein walls is less affected by the non-linear effect of blood denaturalization at higher temperatures, PA thermometry in a squeezed vein (Figure 4c,e) shows a higher accuracy than a catheter located in the uncompressed vessel and surrounded by blood (Figure 4b,d). Figure 4d indicates that the PA amplitude generated at the catheter tip placed in the blood medium follows the temperature variations caused by the CW laser. While PA amplitude changes in the catheter tip placed in the lumen follow the expected trend with the temperature rise, the rate of temperature change measured by the thermocouple is different from PA amplitude change. This could be due to the non-linear change in blood optical absorption at temperatures above 60 °C, which this manuscript discusses later. Figure 4e indicates the change in the temperature-dependent PA signal when the vein is squeezed against the ablation catheter. The rapid increase in the temperature in Figure 4e is due to the high absorption of the 1470 nm CW laser energy by the vein wall [13,54].

The non-linear rise in the PA signal with temperature variations observed in the blood medium during in vivo studies can be explained by the temperature-dependent optical variations at higher temperatures. It is reported in the literature that temperature variations exceeding 50 °C alter the optical absorption properties of tissue chromophores µ_a,_ which affects the capability of PA imaging to guide thermometry procedures [55]. In our internal illumination approach, it is possible to calibrate our thermometry system ex vivo by studying the temperature-dependent absorption properties of tissue chromophores, including blood and water, at higher temperatures, compensating for these variations and providing quantitative temperature measurements during in vivo procedures. The general equation of the PA signal acquired within a blood vessel at the initial temperature can also be defined as $P\left(T_{0}\right)=\Gamma \left(T_{0}\right){µ}_{a}\left(T_{0}\right){\mathrm{Fe}}^{-\alpha_{T_{0}}d}$, where $\alpha_{T_{0}}$ refers to the acoustic attenuation along the ‘d’ direction at the initial temperature. The distance traveled by the acoustic waves from the catheter tip to the US probe is denoted by the term ‘d’. During laser ablation, the tissue temperature increases due to the heat generated by the CW laser from T_0_ (initial temperature) to T_1_ (final temperature). The change in PA amplitude at different temperatures can be defined as $\Delta P\left(T_{0},T_{1}\right)\propto P\left(T_{0}\right)-P\left(T_{1}\right)$. The laser fluence F is constant because the pulsed laser illumination is directly delivered to the target tissue and does not pass through tissue layers. Hence, fluence can be estimated through ex vivo studies. The PA excitation laser pulses interact with a small volume of the tissue at the interface of the ablation catheter, depositing insignificant and minimal extra temperature within the tissue. In addition, the constant tissue pathway between the US transducer and the ablation catheter renders the acoustic attenuation negligible and can be removed, resulting in $\Delta P\left(T_{0},T_{1}\right)\propto \Gamma \left(T_{0}\right)\mu_{a}\left(T_{0}\right)-\Gamma \left(T_{1}\right)\mu_{a}\left(T_{1}\right)$. Quantitative thermometry can be performed by measuring and compensating for the temperature-dependent absorption properties of tissue chromophores by determining the direct relationship between the change in the PA signal and variation in the Gruneisen parameter [47]. This relationship was defined as $\Delta T\propto \Delta \Gamma \left(T_{0},T_{1}\right)=\frac{\Delta P\left(T_{0},T_{1}\right)}{\Delta \mu \left(T_{0},{\;T}_{1}\right)}$. During high-temperature variations (>55 °C), blood flowing within the veins denatures, coagulates, and alters its absorption properties [55]. Previously reported studies have indicated an increase in the absorption coefficient of blood in the temperature interval from 10 to 55 °C [56]. Characterization studies were performed to study the variations in water and blood absorption properties at higher temperatures, ranging from 30 to 85 °C. The optical properties of blood and distilled (DI) water were measured using a custom-designed temperature-controlled spectrophotometer.

**Figure 5:**
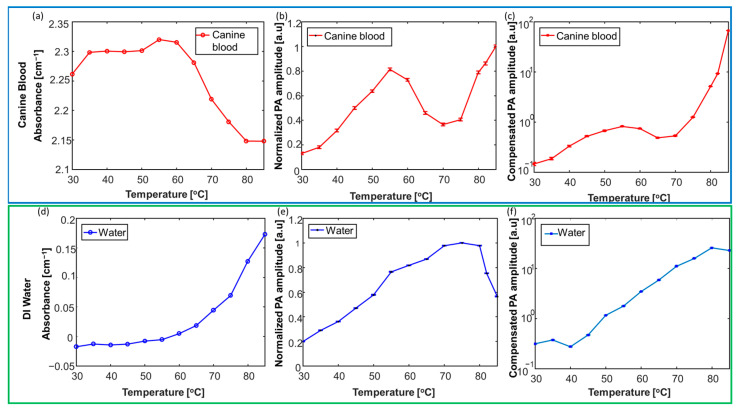
Characterization of the temperature-dependent absorption properties of tissue chromophores, including blood and water. The calibration results of canine blood are highlighted by the top dark-blue panel (**a**–**c**). The calibration results of DI water are highlighted by the top dark-blue panel (**d**–**f**). Variation in the absorbance of (**a**) canine blood and (**d**) DI water is studied at higher temperatures (30–85 °C). Variation of the normalized PA amplitude within (**b**) canine blood and (**e**) DI water mediums within temperatures of 30–85 °C. Compensated PA thermometry measurements at temperatures varied between 30 and 85 °C. The error bars (**b**,**c**,**e**,**f**) represent the standard deviation for each measurement. The image was adapted from [47].

The change in the temperature-dependent absorbance of canine blood is seen in Figure 5. Figure 5a indicated that blood absorption slightly increased within the temperature interval of 30–55 °C. This variation in blood absorption matches the results reported in the previous literature [56]. A rapid decrease in blood absorption was observed until 80 °C, followed by saturation until 85 °C. Figure 5b,e illustrate the variation in the detected PA amplitude without any compensation, normalized to the maximum PA amplitude within the range for blood and water, respectively, with temperature variations. The non-linear temperature-dependent PA signal variation in Figure 5b is due to the altered optical absorption properties induced by blood denaturation and coagulation at a specific temperature interval (>55 °C). The rise in PA signal amplitude at temperatures >70 °C can be explained by changes in the temperature-dependent absorption coefficient and the Gruneisen parameter. However, Figure 5d,e illustrated a linear variation in PA signal in the water medium, dictated by its optical absorption parameter. The compensated PA amplitude described in Figure 5c,f is computed by dividing the normalized PA amplitude by the corresponding absorption coefficient and temperatures. The compensated PA signal function of the Gruneisen parameter is independent of the temperature-dependent variations of the absorption coefficient of the surrounding medium containing the pulsed laser-coupled catheter. Compensated PA thermometry results of canine blood, shown in Figure 5c, indicated an increase in the PA amplitude until a specific temperature of 55 °C, followed by a decrease in the PA amplitude until 65 °C. This trend was followed by a sharp increase in the PA amplitude until 85 °C. The changes in the Gruneisen parameter can account for the increase in the PA signal at temperature >70 °C. Figure 5f highlights the compensated result of DI water, which indicates a linear variation between the PA amplitude and temperature changes.

The accuracy of PA thermometry at various depths and in tissue background was evaluated through an ex vivo study. The study involved a catheter carrying a combined beam (pulsed laser at λ = 532 nm and CW laser at λ = 1470 nm) positioned within water-filled US-transparent tubes sandwiched at different depths (1 to 3 cm) within porcine tissue. The temperature within these tubes was increased from their baseline temperatures (T_0_) to specific temperatures (T_F_) using a CW laser power of 8W as follows: (a) a depth of 1cm and T_0_ (30 °C)–T_F_ (40 °C, 50 °C and 60 °C); (b) a depth of 2 cm and T_0_ (30 °C)–T_F_ (70 °C); and (c) a depth of 3 cm and T_0_ (30 °C)–T_F_ (80 °C). The baseline and specific temperature-dependent PA signals were computed by averaging the PA signals at the catheter tip. These PA signals were then normalized, compensated by the absorption parameter at the corresponding temperatures, and the relative change was computed between them at each depth. The temperature approximated by the relative changes in the PA amplitude is defined as $\mathrm{Estimated}\;\mathrm{Temperature}=0.001517{\left(R_{\mathrm{cpa}}\right)}^{3}-0.197{\left(R_{\mathrm{cpa}}\right)}^{2}+8.68\left(R_{\mathrm{cpa}}\right)-128.8$, where R_cpa_ denotes the relative change in the PA amplitude. The error percentage was defined as $\mathrm{Error}\;\%=\frac{\mathrm{Compensated}\;\mathrm{Temperature}-\mathrm{Expected}\;\mathrm{Temperature}}{\mathrm{Expected}\;\mathrm{Temperature}}\times 100\%$ and is shown in Table 1.

**Figure 6:**
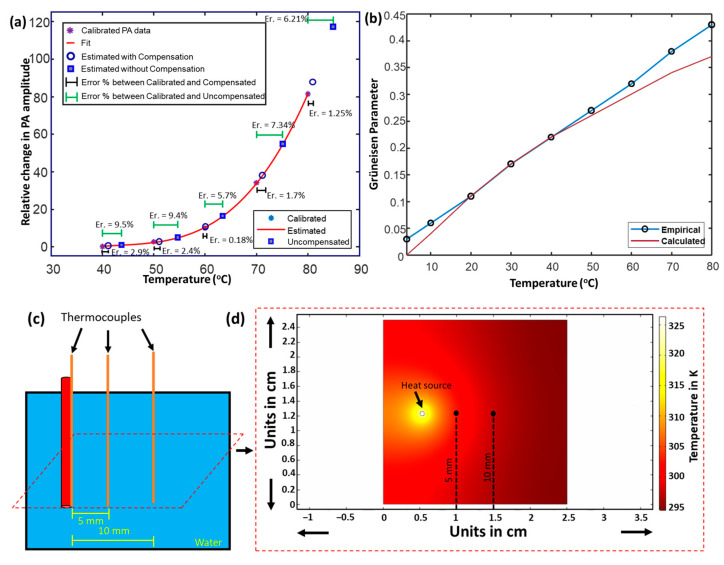
Quantitative PA thermometry and PA-assisted heat diffusion model. (**a**) Polynomial fit curve indicating the calibrated relative changes in the temperature-dependent PA amplitude. The error percentage computed as the difference between the temperature provided by the calibrated and uncompensated PA thermometry is highlighted by horizontal green error bars. The error percentage computed as the difference between the temperature approximated by the calibrated and absorption-compensated PA thermometry is portrayed using black error bars. (**b**) The empirical formula of the Gruneisen parameter of water varies linearly (blue line) within a specific temperature interval (0–80 °C). However, the experimental calculations follow the linear variation (red line) for a particular range of temperatures (20–40 °C). (**c**) Illustration of the experimental study validating the simulated heat diffusion model. (**d**) A simulated heat diffusion model and temperature map indicating the different temperatures around the heat source. The image was adapted from [70].

The temperatures estimated by the compensated PA amplitude (Figure 6a) closely follow the temperature provided by the curve-fitted temperature estimation. Furthermore, the error percentage calculated between the uncompensated data and the calibration curve is roughly five times higher than the error percentage computed from the compensated data. Previously reported studies have indicated a linear variation existing between the Gruneisen parameter (Γ) of water at various temperatures, approximated through the empirical formula [57,58] as $\Gamma \left(T\right)=0.0043+0.0053T$, where T is expressed in °C. However, our studies have validated the linear relationship of the empirical formula [10] only for a specific temperature interval (20–40 °C). This non-linear relationship of the Gruneisen parameter can be explained by the thermal conversion efficiency ηth, which describes the percentage of absorbed photon energy converted into heat [59]. The Gruneisen parameter describes heat conversion efficiency into mechanical energy, so it can be understood that the thermal conversion efficiency indirectly governs the Gruneisen parameter.

Once the local temperature at the ablation catheter tip is measured, it is possible to model the surrounding tissue heating through heat diffusion modeling [60,61]. This temperature map is pivotal in determining the treatment outcome of several image-guided laser ablation procedures [61,62,63]. Through a model-based temperature distribution, the pattern of heat flow from the laser source suspended within a blood vessel can be analyzed [64,65]. A simple heat diffusion model was developed in static conditions to validate the proof-of-concept design of estimating temperatures at different distances of 5 and 10 mm from the heat source. The equation of the heat diffusion model was defined as $\rho_{w}{\;c}_{\mathrm{pw}}\frac{\partial T}{\partial t}=k_{w}\;\nabla^{2}T+Q_{\mathrm{lasersource}}+h\times \left(T_{\mathrm{ext}}-T\right)$, where C_pw_ indicates the water medium’s specific heat capacity (4182 J/kg °C) [66], ρ_w_ represents the density of the water medium (998 kg/m^3^) [67], K_w_ denotes the thermal conductivity of the water (0.598 W/m.K) [68], and ‘h’ indicates the loss due to the convective heat transfer coefficient of air (2.5 W/(m^2^ K) [69]. The CW laser having a power of 40 W was defined as the heat source. The heat diffusion model, whose pictorial representation is highlighted in Figure 6c, was simulated for 10 min, and the temperature profile is indicated in Figure 6d.

Further validation of the phantom studies was performed by simulating a tissue model like that described in Figure 6c. The temperature variations generated by laser ablation were probed by K-type thermocouples positioned at 5 and 10 mm distances for 10 min. The error percentage computed between the simulated and phantom studies is defined as $\mathrm{Error}\;\%=\frac{\mathrm{Experimental}\;\mathrm{Temperature}-\;\mathrm{Approximate}\;\mathrm{Temperature}}{\mathrm{Approximate}\;\mathrm{Temperature}}\times 100\%$. The error percentage computed between the simulated and phantom studies is described in Table 2.

The study revealed good agreement between the experimental data and the simulated temperatures acquired at different distances from the heat source. However, the effect of bubbles generated by the laser energy in water may increase the heat transfer, generating errors between the simulated and the experimental data.

## 4. Integration of Photoacoustic Imaging and Laser Ablation Devices

**Figure 7:**
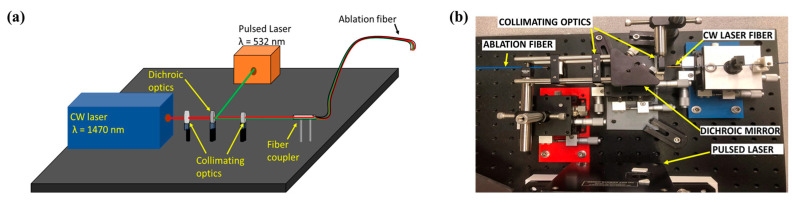
Integration of PA imaging with various laser ablation systems. (**a**) Schematic of integrated PA-guided laser ablation system. (**b**) Photograph of the developed PA-guided laser ablation system. The image was adapted from [47].

Existing image-guided laser ablation devices contain a high-power CW laser for ablation [37]. Translating PA imaging into US-guided devices needs minimal modifications. PA imaging can be integrated into two approaches to a US-image-guided ablation system: external and internal illumination. In the case of internal illumination, the pulsed laser beam can be combined with a CW laser beam through dichroic optics [16]. In the case of external illumination, the pulsed laser-coupled fibers are attached directly to the transducer [57]. The PA-guided laser ablation system for vascular therapies was realized by combining the pulsed laser (λ = 532 nm) and the CW laser (λ = 1470 nm) beams through dichroic optics and suitable collimating optics into an ablation catheter (Figure 7a,b) [17,47]. Compared to methods that use external illumination for thermometry [57,71], internal illumination can provide more accurate PA thermometry as it is free of the limitations imposed by modeling light diffusion through the tissue to account for fluence variations.

## 5. Endoscopic Photoacoustic Imaging for Detecting the Formation and Extension of Thermal Lesions

**Figure 8:**
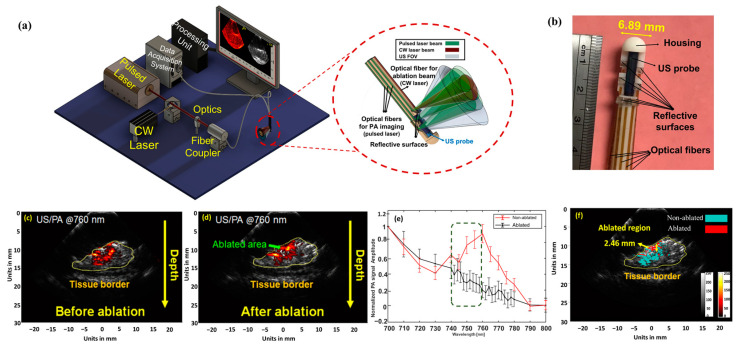
Thermal lesion detection capability of PA imaging: (**a**,**b**) diagram and photograph of the integrated US and PA-guided intracardiac endoscopic and theranostic system. Images were adapted from [73]. PA images of the tissue during (**c**) pre-and (**d**) post-ablation scenarios were obtained at the imaging wavelength of 760 nm. (**e**) Change in the PA amplitude acquired from the tissue surface in the wavelength range between 740 and 760 nm. A distinguishable trend is observed between ablated and healthy tissue regions. (**f**) Each pixel within the ROI was correlated to characterize it as ablated and non-ablated regions. The ablated regions are colored red, and the healthy tissues are indicated using cyan. The image was adapted from [73].

The dependence of the PA signal on the absorption properties of the tissue also allows it to detect the formation of thermal lesions. Several research groups have used PA imaging to detect thermal lesions through different approaches, such as PA spectroscopy [72], dual-wavelength techniques [36], and monitoring the real-time temperature to confirm lesion formation. The PA-based spectroscopy technique distinguishes between different tissue types based on the wavelength-dependent absorption properties of tissue chromophores [73]. Integrated US and PA imaging can be implemented on an intracardiac catheter for monitoring cardiac ablation procedures (Figure 8a) [73]. The PA-guided theranostic endoscopic system for specific cardiac treatments, including atrial fibrillation, was developed by retrofitting a traditional intracardiac echocardiography (ICE) catheter with a pulsed laser light delivery system and an ablation component (Figure 8a) [73]. The pulsed laser light delivery system was realized through six core/cladding optical fibers, and an independent fiber served as the ablation component. The fibers in the pulsed laser light delivery system were arranged specifically to provide optimal tissue illumination. The diameter of the engineered endoscope measured 6.89 mm (Figure 8b).

In a study in which rabbit cardiac tissue was imaged before and after ablation, PA images at a wavelength window of λ = 740–800 nm demonstrated the difference between ablated and non-ablated tissue. Figure 8c,d exhibit the single wavelength approach for λ = 760 nm, an optimal distinction between deoxyhemoglobin and the absorbance of the ablated tissues. The peak in the graph (Figure 8e) at the specified wavelength is exhibited by the absorption spectrum of the healthy tissues. The ablated and healthy regions of the tissue were characterized by applying the Pearson correlation method to the PA signals during pre- and post-ablation scenarios. Every pixel within the lesion, defined by the US image, was analyzed within the known spectrum of deoxyhemoglobin, validating its co-relationship with the healthy or ablated tissue region (Figure 8f).

## 6. Importance of High Temporal Resolution in PA-Guided Ablation Procedures

Some thermal therapies quickly utilize high-power CW laser energy to achieve suitable tissue ablation. During these techniques, the frequently utilized low repetition rate PA-guided system suffers from low temporal resolution and loses accuracy in guiding the catheter tip and thermometry procedures. Inaccuracies induced by low PA imaging frame rates can generate a non-uniform ablation pattern and cause the recurrence of the malady being treated. The low energy of pulsed lasers for integrated PA imaging and ablation catheters provides an opportunity to use higher repetition rate lasers such as diode-pumped solid-state (DPSS) lasers at higher pulse repetition rates [74]. The high repetition rate of PA imaging detected more intermediate positions of the ablation catheter tip, improving the system’s temporal resolution and providing more precise tracking of the ablation catheter tip. We have previously demonstrated using a 500 Hz laser (NL 204-1K-SH, EKSPLA) to enhance PA thermometry accuracy during ablation [74]. The fast PA-guided laser ablation system provided more accurate feedback about the rapid temperature variations in increments of 100 °C per 250 ms. Hence, fast PA imaging can monitor the small-scale temperature variations caused during ablation procedures.

## 7. Future Outlook

Given its notable advantages, such as real-time imaging, US imaging is important in guiding interventional procedures, specifically in guiding ablation catheters and devices. However, its shortcomings, such as sub-optimal contrast and being limited to imaging tissue structure, motivated the medical imaging community to identify alternative or complementary imaging solutions. PA imaging provides accurate real-time device (i.e., ablation catheter) tracking and thermometry. Given the presence of fiber optics in laser ablation devices, integrating these catheters with PA imaging seems to be a viable clinical solution that does not significantly change the current standard of care. Besides providing high-contrast images of ablation catheter devices and creating the potential for accurate thermometry, spectral PA enables the accurate detection of tissue compositions and, thus, the monitoring of the formation of thermal lesions during the procedure. The latter can enhance ablation outcomes and avoid damage to the healthy surrounding tissues. Since laser pulses can be coupled into fibers (as small as 100 microns), PA imaging can also be integrated with other forms of ablations, such as RF and pulsed-field ablation, without redesigning existing devices [75,76,77].

## Figures and Tables

**Table 1:** Validating the precision of PA thermometry in tissue phantoms.

Expected Temperature (°C)	Estimated Temperature (Compensated) (°C)	Estimated Temperature (Un-Compensated) (°C)	Error% Expected Temperature vs. Compensated	Error% Expected Temperature vs. Uncompensated
40	41.19	43.79	2.97%	9.5%
50	51.12	54.71	2.24%	9.42%
60	60.11	63.44	0.18%	5.73%
70	71.19	75.14	1.7%	7.3%
80	81.00	84.97	1.25%	6.2%

**Table 2:** Error percentage computation evaluating the accuracy of the developed heat-diffusion model.

Temperature @ 5mm (°C)		Error %	Temperature @ 10 mm (°C)		Error %
Approx.	Experimental	5.6%	Approx.	Experimental	8.26%
34.23	36.16		31.7	34.32	

## Data Availability

Not applicable.
